# Complications and Laboratory Test Findings Among Patients With Generalized Pustular Psoriasis: A Retrospective Chart Review Study

**DOI:** 10.1111/exd.70227

**Published:** 2026-03-16

**Authors:** Ryuhei Okuyama, Yukari Okubo, Shinichi Imafuku, Yayoi Tada, Keiichi Yamanaka, Kazumitsu Sugiura, Yukie Yamaguchi, Masahito Yasuda, Wataru Sakamoto, Morihisa Saitoh, Akimichi Morita

**Affiliations:** ^1^ Department of Dermatology Shinshu University School of Medicine Matsumoto Japan; ^2^ Department of Dermatology Tokyo Medical University Tokyo Japan; ^3^ Dermatology, Faculty of Medicine Fukuoka University Fukuoka Japan; ^4^ Department of Dermatology Teikyo University School of Medicine Tokyo Japan; ^5^ Department of Dermatology Mie University Graduate School of Medicine Tsu Japan; ^6^ Department of Dermatology Fujita Health University School of Medicine Toyoake Japan; ^7^ Department of Environmental Immuno‐Dermatology Yokohama City University Graduate School of Medicine Yokohama Japan; ^8^ Department of Dermatology Gunma University Graduate School of Medicine Maebashi Japan; ^9^ Nippon Boehringer Ingelheim Tokyo Japan; ^10^ Department of Geriatric and Environmental Dermatology Nagoya City University Graduate School of Medical Sciences Nagoya Japan

**Keywords:** comorbidities, psoriasis, retrospective study, symptom flare‐up

## Abstract

Generalized pustular psoriasis (GPP) is a rare, chronic, inflammatory skin disease characterised by widespread eruption of sterile, macroscopic pustules. Patients with GPP can present with multiple comorbidities that may influence treatment. This study aimed to assess the frequency of psoriasis‐related complications and non–psoriasis‐related comorbidities, and clinical laboratory findings, at the time of GPP diagnosis among patients with GPP. This was a retrospective, longitudinal medical chart review of data from patients with a documented GPP diagnosis attending 29 GPP referral hospitals in Japan. Demographics and clinical characteristics were assessed at baseline (within 6 months prior to and 3 months after GPP diagnosis), including psoriasis‐related complications, non–psoriasis‐related comorbidities, and clinical laboratory findings. Overall, 205 patients with GPP were included; 48.3% were female, and median age at initial diagnosis was 53 years. Similar proportions of patients had mild (36.1%), moderate (30.7%) and severe (33.2%) GPP at baseline, using Japanese Dermatological Association‐GPP severity criteria. Most patients (69.8%) had psoriasis‐related complications at baseline, with the most common being psoriasis vulgaris (42.9%) and psoriatic arthritis (26.8%). Non–psoriasis‐related comorbidities were present in 69.3% of patients with GPP at baseline, with the most common being hypertension (28.3%), dyslipidaemia (16.6%) and diabetes mellitus (16.1%). There was large variability in laboratory test values between patients. These results demonstrated that, at the time of GPP diagnosis, patients with GPP have multiple burdens of both psoriasis‐related complications and non–psoriasis‐related comorbidities.

## 1 Introduction

Generalized pustular psoriasis (GPP) is a rare, chronic, neutrophilic inflammatory skin disease characterised by episodes of widespread eruption of sterile, macroscopic pustules [1, 2]. Not only does GPP affect the skin, but if left untreated, systemic inflammation can cause life‐threatening complications, such as septic shock, capillary leak syndrome, acute respiratory distress syndrome, and cardiac failure [3]. Furthermore, patients with GPP often present with multiple complications and comorbidities that can impact their quality of life and influence the optimal management approach [4].

Among the complications observed in patients with GPP, concomitant psoriasis vulgaris (PsV) and psoriatic arthritis (PsA) occur frequently [5]. Although estimates vary between studies, it has been reported that 42%–85% of GPP cases are preceded by PsV [6, 7, 8, 9], and PsA occurs in 13%–35% of patients with GPP [7, 10, 11, 12, 13, 14, 15]. Furthermore, the presence of PsA is associated with a greater risk of subsequent GPP diagnosis—patients with PsV and comorbid PsA are 20‐fold more likely to be diagnosed with GPP compared with patients who have PsV without PsA [16].

In addition to psoriasis‐related complications, patients with GPP experience a high burden of non–psoriasis‐related comorbidities, including hypertension, diabetes mellitus, and hyperlipidaemia [6, 11, 12, 13, 14, 17, 18, 19]. For example, in a large cohort study from Japan, the proportion of patients with GPP who had these comorbidities was greater than that of matched controls (hypertension: 37% vs. 22%; diabetes mellitus: 15% vs. 7%; hyperlipidaemia: 16% vs. 10%) [12].

Along with prompt treatment of GPP, understanding the range of complications present at the time of GPP diagnosis is important so that patients can be treated in a personalised, holistic manner [4]. However, at present, there are limited data on the occurrence of comorbidities among large patient cohorts with GPP [19]. In Japan, although a few recent studies have provided insight on this topic [11, 12, 17, 18], additional data are needed. As such, the aim of this study was to assess the frequency of psoriasis‐related complications and non–psoriasis‐related comorbidities at the time of GPP diagnosis among patients with GPP and to evaluate clinical laboratory findings in this population using a real‐world retrospective dataset from Japan.

## 2 Methods

### 2.1 Study Design and Patients

The study design has been described in detail previously [18]. Briefly, this was a retrospective, longitudinal review of medical records for patients diagnosed with GPP. Data were included from 29 GPP referral hospitals in Japan. Eligible patients had a documented GPP diagnosis (based on the 2006 Japanese Dermatological Association [JDA] criteria) [4] and ≥ 6 months of continuous observation within 10 years prior to the date of ethics review committee approval at each study site. Patients participating in a clinical trial for medical devices or drugs before or at the time of enrolment were excluded, unless the drugs they received were given at the approved dose and administration.

Written informed consent was obtained from patients or from a designated proxy if the patient was a minor or was unable to provide informed consent. Consent was obtained at a hospital visit prior to registration of the patient in the electronic data capture system. Patients who were unable to visit a hospital due to death or hospital transfer were registered for electronic data capture but were opted out of follow‐up in accordance with Japanese ethical guidelines for clinical studies [20].

The study was conducted in accordance with the Declaration of Helsinki, the Ethical Guidelines for Medical and Health Research Involving Human Subjects, and the Ethical Guidelines for Human Genome and Genetic Analysis Research [21, 22].

### 2.2 Data Collection

Data extraction was initiated after ethics review committee approval at each site and completed by November 2021. Data for eligible patients were extracted from medical records and patient reports submitted annually to the Ministry of Health, Labour and Welfare by the study investigators or research collaborators and entered into the electronic data capture system, which was submitted to the data centre for analysis. Baseline data and data from the follow‐up period were included. Baseline was defined as the date of initial GPP diagnosis. Baseline data were collected during the 6‐month period prior to initial GPP diagnosis; however, if no data were available during this period, data collected within 3 months after GPP diagnosis were used. The follow‐up period started at the date of initial GPP diagnosis and ended at the date of ethics review committee approval for each site.

Details on baseline psoriasis‐related complications and non–psoriasis‐related comorbidities were collected from medical records. To obtain standardised information around psoriasis‐related complications, data collection adopted the format used in the designated intractable disease application documents. Clinical laboratory data generally used in daily practice were also collected. Severity of GPP was determined from JDA‐GPP severity criteria, with total scores of 0–6 classified as mild, 7–10 as moderate and 11–17 as severe [4].

### 2.3 Outcomes

The outcomes assessed in this analysis were patient demographics and clinical characteristics at baseline, including psoriasis‐related complications and non–psoriasis‐related comorbidities and clinical laboratory findings (leukocyte count, erythrocyte sedimentation rate [ESR], C‐reactive protein [CRP], immunoglobulin (Ig)G, IgA, IgM, total protein, serum albumin, serum calcium and calculated serum calcium [calculated using Payne's formula in patients with albumin levels < 4.0 g/dL]) [23]. These outcomes were assessed overall and by GPP severity.

### 2.4 Data Analysis

The full analysis set comprised all patients with a GPP diagnosis. Patient demographics and clinical characteristics (complications, comorbidities and clinical laboratory findings) at baseline were analysed descriptively and reported as mean (standard deviation) or median (interquartile range [IQR], minimum and maximum) for continuous variables and as frequency and percentages for categorical variables. If there were no data available at date of GPP diagnosis, patient data from 6 months before initial GPP diagnosis were extracted. If there were no data before GPP diagnosis, data were collected 3 months post‐GPP.

## 3 Results

### 3.1 Patient Demographics

**FIGURE 1:**
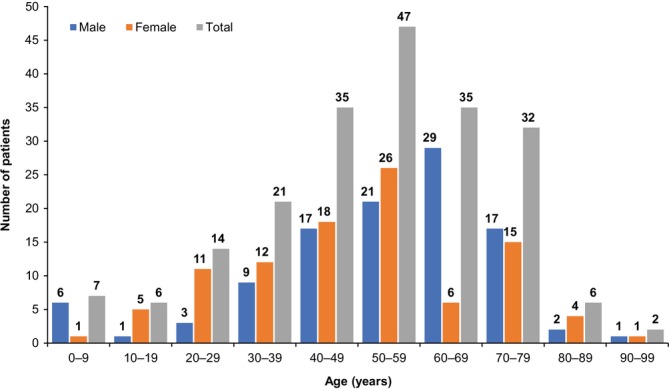
Baseline age and sex distribution in Japanese patients with GPP. Abbreviation: GPP, generalized pustular psoriasis.

Demographics of the 205 patients with GPP included in this chart review have been reported previously [18]. Briefly, 48.3% of patients were female, and the median age at initial diagnosis was 53 years (IQR 42–66), with age distribution concentrated between 40 years and < 80 years (Figure 1). At baseline, similar proportions of patients had mild, moderate and severe GPP (74/205 [36.1%], 63/205 [30.7%] and 68/205 [33.2%], respectively), and 177/205 patients (86.3%) had a GPP flare. The proportion of patients who were hospitalised at initial GPP diagnosis was 35.1% (72/205) among the overall study population and 39.5% (70/177) among those with GPP flare at baseline.

### 3.2 Psoriasis‐Related Complications

**FIGURE 2:**
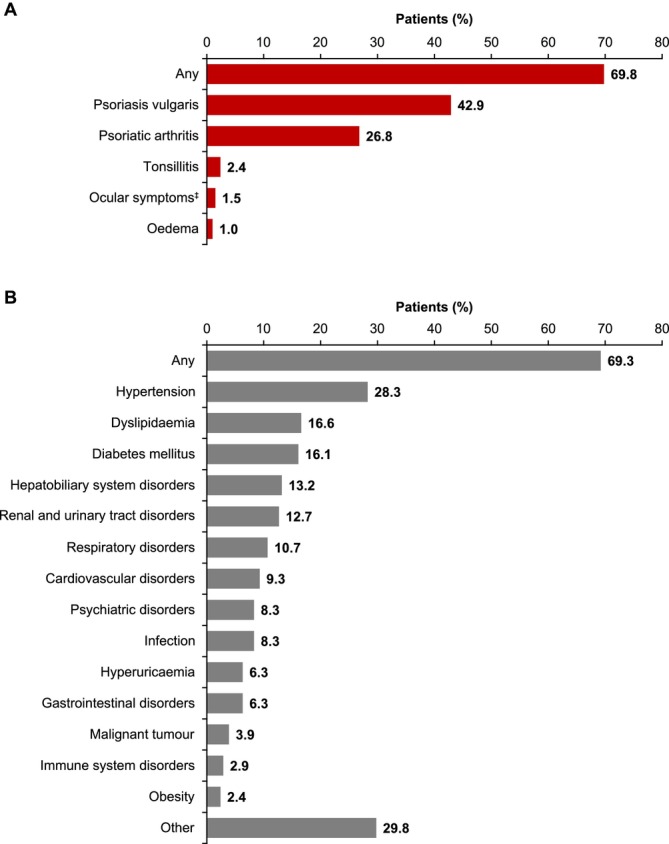
Baseline^†^ concomitant (A) psoriasis‐related complications and (B) non–psoriasis‐related comorbidities in Japanese patients with GPP (*N* = 205). Data represent multiple selections for each patient. Complications/comorbidities occurring in ≥ 1% of patients are shown. ^†^If there were no data available at date of GPP diagnosis, patient data from 6 months before initial GPP diagnosis were extracted. If there were no data before GPP diagnosis, data were collected 3 months post‐GPP diagnosis. ^‡^Keratoconjunctivitis, uveitis, iritis. Abbreviation: GPP, generalized pustular psoriasis.

Over two‐thirds of patients with GPP had psoriasis‐related complications at baseline (143/205 [69.8%]; Figure 2A), with the most common being concomitant PsV (88/205 [42.9%]) followed by PsA (55/205 [26.8%]). Other psoriasis‐related complications were reported by fewer than 3% of patients overall, including tonsillitis (2.4%), ocular symptoms (1.5%) and psoriasis‐related oedema (1.0%). Psoriasis‐related complications at baseline were experienced by similar proportions of patients with mild (53/74 [71.6%]), moderate (44/63 [69.8%]) and severe (46/68 [67.6%]) GPP (Table S1).

### 3.3 Non–Psoriasis‐Related Comorbidities

Non–psoriasis‐related comorbidities were also frequently observed at baseline, occurring in over two‐thirds of patients with GPP (142/205 [69.3%]; Figure 2B). The most frequent non–psoriasis‐related comorbidities (which all occurred in > 10% of patients) were hypertension (58/205 [28.3%]), dyslipidaemia (34/205 [16.6%]), diabetes mellitus (33/205 [16.1%]), hepatobiliary system disorders (27/205 [13.2%]), renal and urinary tract disorders (26/205 [12.7%]) and respiratory disorders (22/205 [10.7%]) (Figure 2B). Other comorbidities (occurring in fewer than 10% of patients) were cardiovascular disorders (9.3%), psychiatric disorders (8.3%), infection (8.3%), hyperuricaemia (6.3%), gastrointestinal disorders (6.3%), malignant tumour (3.9%), immune system disorders (2.9%) and obesity (2.4%). Non–psoriasis‐related comorbidities at baseline were experienced by 51/74 (68.9%), 42/63 (66.7%) and 49/68 (72.1%) patients with mild, moderate and severe GPP, respectively (Table S1).

### 3.4 Clinical Laboratory Results

Clinical laboratory results at baseline for patients with GPP are shown in Table 1. Median values for leukocytes and CRP were above normal reference ranges, and median serum albumin was below normal reference ranges [24].

**TABLE 1:** Baseline^a^ laboratory data in Japanese patients with GPP (*N* = 205).

Laboratory parameter	*n*	Mean (SD)	Median (Q1–Q3)
Leukocytes,^b^ /μL	197	11 915.2 (5480.1)	11 000.0 (7700.0–15 080.0)
ESR, mm/h	128	37.3 (25.5)	34.5 (14.5–54.5)
CRP, mg/dL	191	6.0 (7.2)	2.9 (0.5–9.5)
IgG, mg/dL	126	1108.1 (335.0)	1111.0 (894.0–1301.0)
IgA, mg/dL	120	269.2 (126.7)	247.0 (171.5–347.5)
IgM, mg/dL	112	95.2 (54.1)	80.0 (53.5–125.0)
Total protein, g/dL	182	6.6 (0.9)	6.7 (6.2–7.2)
Serum albumin, g/dL	184	3.5 (0.7)	3.6 (3.0–4.1)
Calculated serum calcium,^c^ mg/dL	157	9.6 (0.6)	9.6 (9.3–9.9)

Abbreviations: CKD‐MBD, Chronic Kidney Disease‐Mineral and Bone Disorder; CRP, C‐reactive protein; ESR, erythrocyte sedimentation rate; GPP, generalized pustular psoriasis; Ig, immunoglobulin; Q, quartile; SD, standard deviation.

^a^ If there were no data available at date of GPP diagnosis, patient data from 6 months before initial GPP diagnosis were extracted. If there were no data before GPP diagnosis, data were collected 3 months post‐GPP diagnosis.

^b^ Data from one patient have not been included as their leukocyte count exceeded 100 000/mL due to having tongue cancer and a thyroid tumour.

^c^ Calcium levels in patients with albumin levels < 4.0 g/dL were calculated using Payne's formula, based on the Clinical Practice Guideline for Management of CKD‐MBD in Japan [23].

As the severity of GPP increased, so did the levels of inflammatory markers, such as leukocytes, ESR and CRP (Table S2). In contrast, levels of IgG, IgA, IgM, total protein and serum albumin decreased with increasing GPP severity (Table S2). Serum calcium was similar between severity groups (mean [standard deviation]: 9.2 mg/dL [0.4], 9.1 mg/dL [0.7] and 8.8 mg/dL [0.7] with mild, moderate and severe GPP, respectively), with a slight decrease with increasing GPP severity. Calculated serum calcium was also similar between severity groups; in contrast with serum calcium, levels of calculated serum calcium did rise very slightly with increasing GPP severity (Table S2).

**FIGURE 3:**
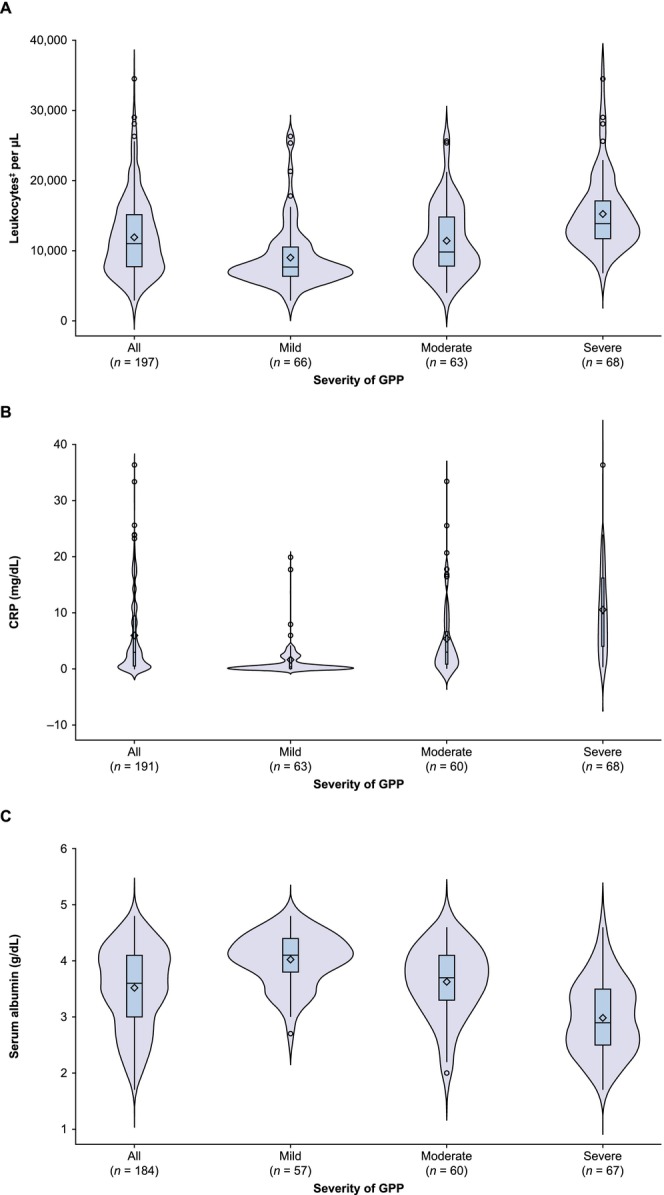
Baseline^†^ laboratory data showing the distribution of (A) leukocytes,^‡^ (B) C‐reactive protein level, and (C) serum albumin level according to disease severity in patients with GPP. The width of each density curve corresponds with the approximate frequency of data points in each region. Box and whisker plots inside each density curve show the median (—), mean (◊), the first and third quartiles (bottom and top of the boxes), and the minimum and maximum values excluding any outliers (the bottom and top whiskers). Circles identify outliers. ^†^If there were no data available at date of GPP diagnosis, patient data from 6 months before initial GPP diagnosis were extracted. If there were no data before GPP diagnosis, data were collected 3 months post‐GPP diagnosis. ^‡^Data from one patient with mild GPP have not been included as their leukocyte count exceeded 100 000/μL due to having tongue cancer and a thyroid tumour. ^§^Determined using JDA‐GPP severity criteria [4], with total scores of 0–6 classified as mild, 7–10 as moderate and 11–17 as severe. Abbreviations: CRP, C‐reactive protein; GPP, generalized pustular psoriasis; JDA, Japanese Dermatological Association.

The distribution of leukocytes (Figure 3A), CRP levels (Figure 3B) and serum albumin levels (Figure 3C) was more concentrated in patients with mild GPP versus moderate or severe GPP. In particular, the distribution of CRP levels in patients with severe GPP was elongated without a distinct peak, compared with the bimodal distribution observed in patients with mild GPP.

## 4 Discussion

To the best of our knowledge, this is the first longitudinal chart review to characterise the high burden of psoriasis‐related complications and non–psoriasis‐related comorbidities, as well as clinical laboratory findings, across GPP severities among patients with GPP at the time of GPP diagnosis. Our findings showed that the most common psoriasis‐related complications at GPP diagnosis were PsV and PsA, and illustrated the high burden of non–psoriasis‐related comorbidities in this population. Our study also highlighted large variability in laboratory values among patients with GPP. In this study, the age group distribution peaked at 50–59 years and was similar to that for patients with GPP and medical expense payments in the Japan Intractable Disease Information Center database [25]. This indicates that the age distribution of our study population was reflective of the national population of patients with GPP in Japan.

Our study found that 26.8% and 42.9% of patients with GPP had comorbid PsA or PsV, respectively. PsV and PsA are well‐recognised comorbidities in patients with GPP, and the systemic autoinflammatory nature of GPP means that comorbidities often include both psoriasis‐related and metabolic/inflammatory (e.g., hypertension and diabetes) conditions. However, our use of psoriasis‐related and non–psoriasis‐related complications aligns with the case report forms used in the study, which were based upon the format used in the designated intractable disease application documents. The proportion of patients with comorbid PsA was higher than that reported for Japanese and US patients with GPP in other studies (12.9%–23.5%) [11, 12, 14, 17]. These studies have also reported wide variations in comorbid PsV and/or other types of psoriasis in patients with GPP, ranging from 6.0% to 65.7% of patients, depending on the categorisation of psoriasis in each study [11, 12, 14, 17]. These findings highlight the variability in the incidence of psoriasis‐related complications in patients with GPP. It has previously been reported that patients with GPP alone have a more severe disease phenotype than those with both GPP and PsV [26], potentially due to a higher frequency of *IL36RN* mutations in those with GPP alone (which may be associated with more severe disease) [10, 27, 28]. In our study, the proportion of patients with GPP who had PsV was broadly similar across subgroups defined by GPP severity, illustrating that patients with GPP and concomitant PsV can have severe symptoms. As reported previously [18], 12/39 (30.8%) of patients with retrospective genetic testing available in our dataset had *IL36RN* mutations. This low number of patients with *IL36RN* mutations means that we cannot reliably assess whether *IL36RN* mutations impacted the risk of comorbidity (or laboratory abnormalities).

Our findings also showed a low occurrence of erythroderma complications. This may reflect the pattern of erythroderma in Japanese patients with GPP, which is not one of a ‘common risk’ for developing an independent complication. Rather, the pattern is that severe GPP flares—especially in genetically predisposed individuals—frequently present with a combination of pustular and erythrodermic features, clinically meeting the diagnostic criteria for both GPP and erythroderma. For this reason, the erythrodermic phenotype can be described as one of the common and characteristic clinical presentations of severe GPP in this specific patient population. The reason pustular psoriasis was listed as a complication was that MedDRA was used for the coding, and the preferred term for ‘Acrodermatitis continua of Hallopeau’ was pustular psoriasis. As this study diagnosed GPP according to the Japanese guidelines criteria, and since ‘Acrodermatitis continua of Hallopeau’ is not considered GPP under these criteria, it is presumed that the clinicians recorded it as a comorbidity.

Non–psoriasis‐related comorbidities were common in this cohort of patients with GPP, with over two‐thirds having at least one comorbidity at GPP diagnosis. The most common non–psoriasis‐related comorbidities were hypertension, dyslipidaemia, diabetes mellitus and hepatobiliary system disorders, confirming findings from previous studies among patients with GPP [11, 12, 14, 17]. In general, there was an increase in the proportion of patients with non–psoriasis‐related comorbidities according to GPP severity, indicating that patients with severe GPP have the highest disease burden. This trend was observed for most comorbidities, including hypertension, dyslipidaemia, cardiovascular diseases, hepatobiliary disorders and infections. It is difficult to distinguish whether the increased rates of cardiovascular disease and hypertension were a result of increased systemic inflammation from GPP or whether these developed as unconnected comorbidities.

The JDA‐GPP guidelines contain recommendations for managing and treating psoriasis‐related complications, such as joint and eye symptoms, and highlight treatments that should be avoided in patients with certain complications or that should be used with caution [4]. However, they do not include guidance for other common non–psoriasis‐related comorbidities, such as hypertension, dyslipidaemia and diabetes mellitus. Given the large proportion of patients with GPP who had non–psoriasis‐related comorbidities at GPP diagnosis in our study, there is a potential need for comprehensive, individualised treatment of patients with GPP that accounts for the various comorbidities that may be present. Some GPP treatment options (e.g., cyclosporine, methotrexate and tumour necrosis factor‐α inhibitors) may worsen comorbidities, such as cardiovascular disease and hepatic function in patients with GPP [4, 29, 30]. As such, the presence of certain comorbidities may limit GPP treatment options, as physicians must consider whether treatment‐associated adverse events may exacerbate these conditions. Timely and effective treatment is vital in controlling disease symptoms in GPP [1], and in patients whose treatment options are limited by the presence of comorbidities, it may take longer to minimise disease severity and decrease the overall burden of GPP. Future research should investigate the efficacy and safety of GPP treatments in patients with commonly reported complications so that guidelines can confidently direct and support physicians in selecting treatment pathways for different populations of patients with GPP.

The clinical laboratory findings in this study showed that as GPP severity increased, levels of laboratory markers of inflammation (leukocytes, ESR and CRP) also increased, whereas levels of serum albumin decreased. This finding is expected given that the criteria used to categorise GPP severity in this study included assessment of laboratory parameters and assigned a greater severity score to patients with higher white blood cell counts, higher CRP levels and lower albumin levels [4]. In our study, serum calcium levels were not high. We adjusted the calcium concentrations in patients with albumin levels < 4.0 g/dL using Payne's formula, based on the Clinical Practice Guideline for Management of Chronic Kidney Disease‐Mineral and Bone Disorder in Japan [23]. Albumin concentration is typically decreased in patients with severe GPP [4, 31], and approximately 45% of serum calcium is bound to albumin [32, 33]. Accordingly, physicians should consider assessing serum albumin–corrected calcium concentrations when managing patients with GPP [23]. Of note, the laboratory findings from this study demonstrate that there is large variability between patients. In particular, the distribution of CRP within each GPP severity grade illustrated a wide range of values between patients, along with outliers. This variability should be taken into consideration when managing patients with GPP and highlights the need to individualise treatment according to individual laboratory findings.

Strengths and limitations for this retrospective chart review have been previously discussed in detail [18]. A key strength is that as GPP is a rare disease, a retrospective chart review allowed for a larger population to be studied compared with that of a clinical trial and allowed for observations in a real‐world context. For this analysis, a limitation is that the baseline period spanned from 6 months before diagnosis to 3 months after diagnosis and, as such, the baseline data on complications, comorbidities and laboratory assessments may not have been collected at the time of GPP diagnosis. For any data collected after GPP diagnosis, it is possible that treatments given at the time of GPP diagnosis may have influenced the findings.

## 5 Conclusions

Our study has demonstrated that at the time of GPP diagnosis, patients with GPP have a high burden of both psoriasis‐related complications and non–psoriasis‐related comorbidities. In addition, we found that there is high variability among clinical laboratory findings at GPP diagnosis. Understanding patient complications, comorbidities and laboratory test trends at GPP diagnosis is important to help guide selection of appropriate, comprehensive treatment plans for patients with GPP. Guidance on the management of GPP in the setting of specific complications will allow personalised GPP treatment plans to be developed.

## Author Contributions

Study design: Akimichi Morita, Morihisa Saitoh, Ryuhei Okuyama, Shinichi Imafuku, Wataru Sakamoto and Yukari Okubo. Study investigator: All authors. Enrolled patients: All authors. Collection and assembly of data: Morihisa Saitoh and Yukie Yamaguchi Data analysis: Morihisa Saitoh, Ryuhei Okuyama and Wataru Sakamoto Data interpretation: All authors. Manuscript preparation: Akimichi Morita, Morihisa Saitoh, Ryuhei Okuyama, Wataru Sakamoto and Yukari Okubo. Manuscript review and revisions: All authors. Final approval of manuscript: All authors.

## Funding

This study was supported by Nippon Boehringer Ingelheim.

## Disclosure

Institutional Review Board Statement: The study protocol was approved by the ethics review committee of each study site, as previously published in Morita et al. [18].

## Consent

Written informed consent was obtained from patients or from a designated proxy if the patient was a minor or was unable to provide informed consent. Consent was obtained at a hospital visit prior to registration of the patient in the electronic data capture system. Patients who were unable to visit a hospital due to death or hospital transfer were registered for electronic data capture but were opted out of follow‐up.

## Conflicts of Interest

The authors met criteria for authorship as recommended by the International Committee of Medical Journal Editors (ICMJE). The authors did not receive payment related to the development of this manuscript. Nippon Boehringer Ingelheim was given the opportunity to review the manuscript for medical and scientific accuracy, as well as intellectual property considerations. Ryuhei Okuyama declares research grants, consulting fees and/or speaker's fees from AbbVie, Amgen, Boehringer Ingelheim, Eisai, Eli Lilly, Janssen, Kyowa Kirin, LEO Pharma, Maruho, Mitsubishi Tanabe, Novartis, Pfizer, Sun Pharmaceutical Industries, Taiho Pharmaceutical, Torii Pharmaceutical and UCB. Yukari Okubo declares grants or contracts from Eisai, Maruho Pharmaceutical, and Shiseido Torii; consulting fees from AbbVie, Amgen, Boehringer Ingelheim, Bristol Myers Squibb, Celgene, Eisai, Eli Lilly, Janssen, JIMRO, Kyowa Kirin, LEO Pharma, Maruho Pharmaceutical, Mitsubishi Tanabe, Novartis, Pfizer, Sanofi, Sun Pharmaceutical Industries, Taiho Pharmaceutical, Torii Pharmaceutical and UCB. Shinichi Imafuku has served as a consultant and/or paid speaker for and/or accepted a research grant from and/or participated in clinical trials sponsored by companies including AbbVie, Amgen, Boehringer Ingelheim, Daiichi Sanyo, Eisai, Eli Lilly, GSK, Janssen, Kyowa Kirin, LEO Pharma, Maruho Pharmaceutical, Novartis, Sun Pharmaceutical Industries, Taiho Yakuhin Kogyo, Torii Yakuhin and UCB. Yayoi Tada declares honoraria and/or grants from AbbVie, Boehringer Ingelheim, Eisai, Eli Lilly, Janssen, Kyowa Kirin, LEO Pharma, Maruho, Mitsubishi Tanabe, Novartis Pharma, Sun Pharmaceutical Industries, Taiho Pharmaceutical, Torii Pharmaceutical and UCB. Keiichi Yamanaka declares research speaker fees and chair fees from Boehringer Ingelheim. Kazumitsu Sugiura declares consulting and/or speaker fees from AbbVie GK, Amgen, Bristol Myers Squibb, Eisai, Eli Lilly Japan, Janssen Pharmaceutical, Kaken Pharmaceutical, Kyowa Kirin, LEO Pharma Japan, Maruho, Minophagen Pharmaceutical, Nippon Boehringer Ingelheim, Nippon Zoki Pharmaceutical, Novartis Pharma, Taiho Pharmaceutical, Tsumura, Sanofi, Sato Pharmaceutical, Sun Pharmaceutical Industries, Torii Pharmaceutical and UCB. Yukie Yamaguchi declares research grants, and/or consulting fees, and/or speaker fees from AbbVie, Amgen, Astellas, Boehringer Ingelheim, Eisai, Eli Lilly, Janssen, Kyowa Kirin, LEO Pharma, Maruho, Mitsubishi Tanabe, Novartis, Sun Pharmaceutical Industries, Taiho Pharmaceutical, Torii Pharmaceutical and UCB Japan. Masahito Yasuda declares receiving consulting fees and/or speaker fees from Boehringer Ingelheim, Janssen, Novartis and Taiho Pharmaceutical, and participating in clinical trials sponsored by Eli Lilly. Wataru Sakamoto and Morihisa Saitoh are employees of Nippon Boehringer Ingelheim. Akimichi Morita declares receiving research grants, consulting fees and/or speaker fees from AbbVie, Boehringer Ingelheim, Eisai, Eli Lilly, Janssen, Kyowa Kirin, LEO Pharma, Maruho, Mitsubishi Tanabe, Nichi‐Iko, Nippon Kayaku, Novartis, Sun Pharmaceutical Industries, Taiho Pharmaceutical, Torii Pharmaceutical and Ushio. Akimichi Morita is also Editor‐in‐Chief of *Experimental Dermatology* and a co‐author of this article. To minimise bias, they were excluded from all editorial decision‐making related to the acceptance of this article for publication.

## Supporting information


**TABLE S1:** Baseline^†^ concomitant psoriasis‐related complications and non–psoriasis‐related comorbidities according to disease severity^‡^ in Japanese patients with GPP.
**TABLE S2:** Baseline^†^ laboratory data according to disease severity^‡^ in Japanese patients with GPP.

## Data Availability

To ensure independent interpretation of clinical study results and enable authors to fulfil their role and obligations under the ICMJE criteria, Boehringer Ingelheim grants all external authors access to relevant clinical study data. In adherence with the Boehringer Ingelheim Policy on Transparency and Publication of Clinical Study Data, scientific and medical researchers can request access to clinical study data, typically 1 year after the approval has been granted by major Regulatory Authorities or after termination of the development programme. Researchers should use the https://vivli.org link to request access to study data and visit https://www.mystudywindow.com/msw/datasharing for further information.
