# Supplementary material for: Complications and Laboratory Test Findings Among Patients With Generalized Pustular Psoriasis: A Retrospective Chart Review Study
Source: Exp Dermatol. 2026 Mar 16;35(3):e70227. doi: 10.1111/exd.70227 (PMC12992671; doi:10.1111/exd.70227)
Supplement: Supplementary file 1 — TABLE S1: Baseline† concomitant psoriasis‐related complications and non–psoriasis‐related comorbidities according to disease severity‡ in Japanese patients with GPP. TABLE S2: Baseline† laboratory data according to disease severity‡ in Japanese patients with GPP. [file EXD-35-e70227-s001.docx]

# SUPPORTING INFORMATION

# Complications and Laboratory Test Findings Among Patients With Generalized Pustular Psoriasis: A Retrospective Chart Review Study

Ryuhei Okuyama, Yukari Okubo, Shinichi Imafuku, Yayoi Tada, Keiichi Yamanaka, Kazumitsu Sugiura, Yukie Yamaguchi, Masahito Yasuda, Wataru Sakamoto, Morihisa Saitoh, Akimichi Morita

**TABLE S1** Baseline^a^ concomitant psoriasis-related complications and non–psoriasis-related comorbidities according to disease severity^b^ in Japanese patients with GPP.

| ***n* (%)** | **GPP severity** | | | | |
| --- | --- | --- | --- | --- | --- |
|  | **Mild**  **(*n* = 74)** | **Moderate**  **(*n* = 63)** | | **Severe**  **(*n* = 68)** | **All**  **(*N* = 205)** |
| **Psoriasis-related complications** | | | | | |
| **Any** | **53 (71.6)** | | **44 (69.8)** | **46 (67.6)** | **143 (69.8)** |
| **Occurring in ≥ 1% of all patients:** |  | |  |  |  |
| Psoriasis vulgaris | 30 (40.5) | | 32 (50.8) | 26 (38.2) | 88 (42.9) |
| Psoriatic arthritis | 23 (31.1) | | 18 (28.6) | 14 (20.6) | 55 (26.8) |
| Tonsillitis | 2 (2.7) | | 2 (3.2) | 1 (1.5) | 5 (2.4) |
| Ocular symptoms^c^ | 0 (0.0) | | 1 (1.6) | 2 (2.9) | 3 (1.5) |
| Oedema | 0 (0.0) | | 0 (0.0) | 2 (2.9) | 2 (1.0) |
| **Occurring in < 1% of all patients:** |  | |  |  |  |
| Epicondylitis | 1 (1.4) | | 0 (0.0) | 0 (0.0) | 1 (0.5) |
| Pustular psoriasis | 1 (1.4) | | 0 (0.0) | 0 (0.0) | 1 (0.5) |
| Palmoplantar pustulosis**^d^** | 1 (1.4) | | 0 (0.0) | 0 (0.0) | 1 (0.5) |
| Pigmentation disorder | 1 (1.4) | | 0 (0.0) | 0 (0.0) | 1 (0.5) |
| Tenosynovitis | 1 (1.4) | | 0 (0.0) | 0 (0.0) | 1 (0.5) |
| Tendinopathy | 1 (1.4) | | 0 (0.0) | 0 (0.0) | 1 (0.5) |
| Erythroderma | 0 (0.0) | | 1 (1.6) | 0 (0.0) | 1 (0.5) |
| Cellulitis | 0 (0.0) | | 0 (0.0) | 1 (1.5) | 1 (0.5) |
| Fungal infection | 0 (0.0) | | 0 (0.0) | 1 (1.5) | 1 (0.5) |
| Nasopharyngitis | 0 (0.0) | | 0 (0.0) | 1 (1.5) | 1 (0.5) |
| Pharyngitis | 0 (0.0) | | 0 (0.0) | 1 (1.5) | 1 (0.5) |
| Contact dermatitis | 0 (0.0) | | 0 (0.0) | 1 (1.5) | 1 (0.5) |
| **Non–psoriasis-related comorbidities** | | | | | |
| **Any** | **51 (68.9)** | | **42 (66.7)** | **49 (72.1)** | **142 (69.3)** |
| **Occurring in ≥ 1% of all patients:** |  | |  |  |  |
| Hypertension | 13 (17.6) | | 21 (33.3) | 24 (35.3) | 58 (28.3) |
| Dyslipidaemia | 9 (12.2) | | 11 (17.5) | 14 (20.6) | 34 (16.6) |
| Diabetes mellitus | 11 (14.9) | | 8 (12.7) | 14 (20.6) | 33 (16.1) |
| Hepatobiliary system disorders | 5 (6.8) | | 13 (20.6) | 9 (13.2) | 27 (13.2) |
| Renal and urinary tract disorders | 6 (8.1) | | 5 (7.9) | 15 (22.1) | 26 (12.7) |
| Respiratory disorders | 8 (10.8) | | 5 (7.9) | 9 (13.2) | 22 (10.7) |
| Cardiovascular disorders | 3 (4.1) | | 7 (11.1) | 9 (13.2) | 19 (9.3) |
| Psychiatric disorders | 6 (8.1) | | 4 (6.3) | 7 (10.3) | 17 (8.3) |
| Infection | 5 (6.8) | | 5 (7.9) | 7 (10.3) | 17 (8.3) |
| Hyperuricaemia | 2 (2.7) | | 5 (7.9) | 6 (8.8) | 13 (6.3) |
| Gastrointestinal disorders | 4 (5.4) | | 3 (4.8) | 6 (8.8) | 13 (6.3) |
| Malignant tumour^e^ | 4 (5.4) | | 1 (1.6) | 3 (4.4) | 8 (3.9) |
| Immune system disorders | 4 (5.4) | | 1 (1.6) | 1 (1.5) | 6 (2.9) |
| Obesity | 1 (1.4) | | 1 (1.6) | 3 (4.4) | 5 (2.4) |
| **Other^f^** | 23 (31.1) | | 17 (27.0) | 21 (30.9) | 61 (29.8) |

Abbreviations: GPP, generalized pustular psoriasis; JDA, Japanese Dermatological Association.

Data represent multiple selections for each patient.

^a^ If there were no data available at date of GPP diagnosis, patient data from 6 months before initial GPP diagnosis were extracted. If there were no data before GPP diagnosis, data were collected 3 months post-GPP diagnosis.

^b^ Determined using JDA-GPP severity criteria,^1^ with total scores of 0–6 classified as mild, 7–10 as moderate and 11–17 as severe.

^c^ Keratoconjunctivitis, uveitis, iritis.

^d^ Although rare, there are cases where palmoplantar pustulosis precedes the onset of GPP, and this case was considered to be one of those.

^e^ Malignant tumours in eight patients included prostate cancer (n=3), breast cancer (n=2), oesophageal carcinoma (n=1), tongue neoplasm malignant stage unspecified (n=1), and bladder cancer and thyroid cancer in the same patient.

^f^ Other comorbidities: osteoporosis (*n* = 7 [3.4%]); cataract (*n* = 6 [2.9%]); seasonal allergy (*n* = 5 [2.4%]); anaemia, dermatitis atopic, glaucoma and intervertebral disc protrusion (*n* = 3 [1.5%] each); back pain, Basedow’s disease, constipation, epilepsy, goiter, hypoalbuminaemia, oedema, spinal compression fracture, uterine leiomyoma and fibromyalgia (*n* = 2 [1.0%] each); abdominal mass, acne, ankylosing spondylitis, astigmatism, blepharospasm, cerebral infarction, chest X-ray abnormal, chronic tonsillitis, conjunctivitis allergic, dementia, dermatitis, dizziness, dysmenorrhea, eczema asteatotic, folate deficiency, gestational diabetes, hemorrhoids, hyperkalaemia, hypothyroidism, ingrowing nail, insomnia, iron deficiency anaemia, Ménière's disease, migraine, mixed connective tissue disease, moyamoya disease, myelopathy, neoplasm skin, neuropathy peripheral, normochromic normocytic anaemia, osteoarthritis, polycystic ovaries, pruritus, rhinitis allergic, rosacea, skin papilloma, systemic lupus erythematosus, thyroiditis subacute, Turner's syndrome, urticaria, vitreous floaters, deafness unilateral, ocular vascular disorder, adrenal mass, allergy to plants, asteatosis, thyroid mass, hot flush, genital hemorrhage, sudden hearing loss, spondylolisthesis, rhegmatogenous retinal detachment, spinal stenosis and immune thrombocytopenia (*n* = 1 [0.5%] each).

**TABLE S2** Baseline^a^ laboratory data according to disease severity^b^ in Japanese patients with GPP.

| **Laboratory parameter** | **GPP severity** | | |
| --- | --- | --- | --- |
|  | **Mild (*n* = 74)** | **Moderate (*n* = 63)** | **Severe (*n* = 68)** |
| Leukocytes,^c^ /μL |  | | |
| *n* | 66 | 63 | 68 |
| Mean (SD) | 9031.7 (4546.8) | 11 388.7 (4957.0) | 15 201.8 (5064.0) |
| Median (Q1–Q3) | 7670.0 (6370.0–10 500.0) | 9800.0 (7800.0–14 800.0) | 13 860.0 (11 700.0–17 100.0) |
| ESR, mm/hr |  | | |
| *n* | 38 | 41 | 49 |
| Mean (SD) | 29.3 (24.3) | 31.2 (22.0) | 48.4 (25.6) |
| Median (Q1–Q3) | 24.5 (10.0–41.0) | 30.0 (13.0–42.0) | 51.0 (28.0–64.0) |
| CRP, mg/dL |  | | |
| *n* | 63 | 60 | 68 |
| Mean (SD) | 1.7 (3.5) | 5.4 (6.9) | 10.6 (7.5) |
| Median (Q1–Q3) | 0.4 (0.1–2.0) | 3.0 (0.9–6.7) | 9.8 (4.1–16.3) |
| IgG, mg/dL |  | | |
| *n* | 32 | 45 | 49 |
| Mean (SD) | 1269.7 (326.5) | 1082.1 (304.3) | 1026.5 (337.3) |
| Median (Q1–Q3) | 1240.0 (1077.5–1405.0) | 1085.0 (935.0–1237.0) | 967.0 (761.0–1230.0) |
| IgA, mg/dL |  | | |
| *n* | 32 | 41 | 47 |
| Mean (SD) | 290.6 (124.4) | 261.5 (137.3) | 261.3 (119.4) |
| Median (Q1–Q3) | 303.0 (209.5–356.5) | 230.0 (175.0–343.0) | 219.0 (159.0–332.0) |
| IgM, mg/dL |  | | |
| *n* | 31 | 38 | 43 |
| Mean (SD) | 110.9 (56.5) | 102.9 (64.5) | 77.0 (34.7) |
| Median (Q1–Q3) | 99.0 (67.0–140.0) | 82.0 (53.0–139.0) | 67.0 (51.0–106.0) |
| Total protein, g/dL |  | | |
| *n* | 59 | 56 | 67 |
| Mean (SD) | 7.1 (0.6) | 6.7 (0.8) | 6.2 (0.9) |
| Median (Q1–Q3) | 7.1 (6.8–7.5) | 6.8 (6.3–7.2) | 6.3 (5.7–6.7) |
| Serum albumin, g/dL |  | | |
| *n* | 57 | 60 | 67 |
| Mean (SD) | 4.0 (0.5) | 3.6 (0.6) | 3.0 (0.7) |
| Median (Q1–Q3) | 4.1 (3.8–4.4) | 3.7 (3.3–4.1) | 2.9 (2.5–3.5) |
| Calculated serum calcium,^d^ mg/dL |  | | |
| *n* | 44 | 48 | 65 |
| Mean (SD) | 9.3 (0.5) | 9.6 (0.5) | 9.8 (0.7) |
| Median (Q1–Q3) | 9.3 (9.0–9.6) | 9.6 (9.3–9.8) | 9.8 (9.5–10.1) |

Abbreviations: CKD-MBD, Chronic Kidney Disease-Mineral and Bone Disorder; CRP, C-reactive protein; ESR, erythrocyte sedimentation rate; GPP, generalized pustular psoriasis; Ig, immunoglobulin; JDA, Japanese Dermatological Association; Q, quartile; SD, standard deviation.

^a^ If there were no data available at date of GPP diagnosis, patient data from 6 months before initial GPP diagnosis were extracted. If there were no data before GPP diagnosis, data were collected 3 months post-GPP diagnosis.

^b^ Determined using JDA-GPP severity criteria [1], with total scores of 0–6 classified as mild, 7–10 as moderate and 11–17 as severe.

^c^ Data from one patient with mild GPP has not been included as their leukocyte count exceeded 100 000/mL due to having tongue cancer and a thyroid tumour.

^d^ Calcium levels in patients with albumin levels < 4.0 g/dL were calculated using Payne’s formula, based on the Clinical Practice Guideline for Management of CKD-MBD in Japan [2].

**References**

1. H. Fujita, T. Terui, K. Hayama, et al., “Japanese Guidelines for the Management and Treatment of Generalized Pustular Psoriasis: The New Pathogenesis and Treatment of GPP,” *Journal of Dermatolology* 45, no. 11 (2018): 1235–1270.

2. M. Fukagawa, K. Yokoyama, F. Koiwa, et al., “Clinical Practice Guideline for the Management of Chronic Kidney Disease-Mineral and Bone Disorder,” *Therapeutic Apheresis and Dialysis* 17, no. 3 (2013): 247–288.
